# “Saint Google, now we have information!”: a qualitative study on narratives of trust and attitudes towards maternal vaccination in Mexico City and Toluca

**DOI:** 10.1186/s12889-021-11184-y

**Published:** 2021-06-18

**Authors:** Clarissa Simas, Heidi J. Larson, Pauline Paterson

**Affiliations:** 1 Department of Infectious Disease Epidemiology, London School of Hygiene & Tropical Medicine, Keppel Street, London, WC1E 7HT UK; 2 Department of Health Metrics Sciences, University of Washington, Seattle, USA; 3 National Institute for Health Research Health Protection Research Unit (NIHR-HPRU) in Immunisation, London School of Hygiene and Tropical Medicine in partnership with Public Health England, London, UK

**Keywords:** Maternal immunization, Vaccine confidence, Information, Rumours, Trust, Pregnant women

## Abstract

**Background:**

Maternal vaccination is key to decreasing maternal and infant mortality globally. Yet perceptions about maternal vaccines and immunization among pregnant women are often understudied, particularly in low- and middle- income countries. This qualitative study explored trust, views, and attitudes towards maternal immunization among pregnant women in Mexico. A total of 54 women from Mexico City and Toluca participated in the in-depth interviews and focus groups. We explored participants’ experiences with maternal vaccination, as well as how they navigated the health system, searched for information, and made decisions around maternal immunization.

**Results:**

Our findings point to issues around access and quality of maternal healthcare, including immunizations services. While healthcare professionals were recognized for their expertise, participants reported not receiving enough information to make informed decisions and used online search engines and digital media to obtain more information about maternal healthcare. Some participants held strong doubts over the benefits of vaccination and were hesitant about the safety and efficacy of maternal vaccines. These concerns were also shared by pregnant women who had been vaccinated. Some participants disclosed low levels of trust in government and vaccination campaigns.

**Conclusion:**

Pregnant women, soon to be parents and making vaccination decisions for their child, constitute an important target group for policymakers seeking optimal maternal as well as childhood immunization coverage. Our findings highlight the importance of targeted communication, trust-building and engagement strategies to strengthen confidence in immunization amongst this group.

**Supplementary Information:**

The online version contains supplementary material available at 10.1186/s12889-021-11184-y.

## Introduction

Maternal vaccination is key to reducing maternal and infant mortality worldwide [1]. Maternal immunization can reduce the incidence of influenza, pertussis and tetanus in both mothers and neonates [2]. Yet the views and attitudes of pregnant women towards maternal immunization are often understudied, particularly in low- and middle- income settings [2]. Insights into the drivers of vaccine hesitancy among pregnant women can help inform interventions to encourage uptake of vaccines.

Current vaccination coverage rates in Latin America and the Caribbean fall short of the targets set by the Pan American Health Organization, with coverage varying both between and within countries [3, 4]. Coverage rates for vaccines recommended during pregnancy tend to fluctuate over time [5]. In Mexico, for example, influenza vaccination rates for pregnant women fell from 81% in 2018 [5] to 65% in 2020 [6]; while Tdap coverage for pregnant women jumped from 52% in 2015 to 96% in 2016 before dropping again to 60% in 2018. Despite inadequate coverage rates, a recent systematic review of barriers to vaccination in Latin America [7], found very few studies which have looked at barriers to vaccination among pregnant women in the region [8–10].

### Mexico’s health reform and current maternal health and immunization services

Starting in the late 1980s, several Latin American countries launched social sector reforms to decrease poverty and lessen inequalities [11]. In this period, Mexico embarked on a structural health system reform aimed at improving health outcomes for lower-income populations, which are often excluded from healthcare services. At the center of these reforms was the so-called Popular Health Insurance, or *Seguro Popular,* a subsidized insurance-based system offering free access to an explicit set of healthcare interventions. While these efforts contributed to reducing barriers to access healthcare, this was a segregated system comprised of a well-resourced health insurance scheme for salaried workers and their families and a popular health insurance serving poor and vulnerable individuals. The latter had lower standards of quality and required payment at point of service [12].

Currently, Mexico maintains this fragmented healthcare system with three main types of health insurance, each of which caters to a different strata of the Mexican population: first, state health insurance for those employed; second, private health services providers with their own clinics and hospitals for those who can afford; and government-sponsored social insurance for those not covered by previous schemes [13]. This segregation in healthcare access and quality is a barrier to fulfilling the right to health and is linked to increased inequalities in access [12].

The Mexican public health system is overstretched and healthcare quality remains unsatisfactory for most of the population [14]. Private medical care remains better equipped to provide specialized procedures and generally has higher quality of care [13], although this can be variable across providers [14]. In one survey, private institutions were overall ranked positively by users in the country [14].

In an effort to decrease maternal and neonate mortality, Mexico partially restructured its health systems to provide free prenatal care, through *Seguro Popular* [15]. While studies have reported on this effort to reduce maternal morbidity [16], little is known about confidence in maternal immunization among pregnant Mexican women [17].

### Dimensions of trust associated with vaccination

Vaccine hesitancy, a common barrier to vaccination [18], has been defined by the Strategic Advisory Group of Experts (SAGE) working group as a spectrum that goes from delay in acceptance to outright refusal of vaccination, despite the availability of immunization services [19]. Vaccine hesitancy is influenced by complacency (perception there is no value or need for a vaccine), convenience (financial and geographical barriers to access or absence of vaccination services) and confidence (related to lack of trust in the vaccine, provider and/or policy). Confidence represents trust in the effectiveness and safety of vaccines, in the system that delivers them (including reliability of healthcare professionals), and trust in policy makers who decide on the need for vaccines. Vaccine confidence is also determined by trust in the wider health system, an important factor which influences the vaccine decision-making process [20].

Trust is multidimensional and occurs within relationships and interactions with systems. Trust stems from beliefs about how the other will act and this belief, in turn, determines whether a person is willing to accept the other person’s advice [21]. To trust is a choice and trust-based collaboration is built on believing the other party will act in someone’s best interest [20]. Trust is necessary for cooperation when there is uncertainty and, at times, trust can be described as a leap of faith which takes place through social relationships and within social political contexts [22].

Different dimensions of trust are associated with the decision to vaccinate. In this article, our conceptualization of trust in vaccines has emerged from a systematic literature review measuring trust in vaccination [20]. Vaccine acceptance involves trust in the wider healthcare system, trust in government and political system, trust in the safety and efficacy of vaccines, as well as trust in the providers who administer them [20]. In the context of vaccine decisions, trust becomes important in helping to make a risk/benefit assessment [20].

Trust in vaccines reflects historical legacies of trust and mistrust with institutions and health actors. These varied narartives and experiences of individuals highlight how public trust in vaccines and immunization programmes is highly variable and locally specific [20]. In addition, trust in information about vaccines depends not only on the trust of information itself, but also the trust in the source of that information. To that end, we considered trust in information as nested within the trust held in the source of that information [20].

Recognizing trust as a complex web of vaccine-related factors can provide valuable insights into the levers of vaccine acceptance, hesitancy or refusal [20]. This study explore narratives of trust, views and attitudes towards maternal immunization among pregnant women in Mexico.

## Methods

### Study site

This study was conducted in Toluca and Mexico City, Mexico. Both cities are among the most populous in Mexico, but with marked differences in the use, offer and configuration of health services, particularly in areas away from the main financial hub in Mexico City. The cities are interconnected, with regular movement of people from one to the other, and equally have high urban demographic concentration – all of which can facilitate vaccine preventable disease outbreaks in areas of low vaccine coverage. This article adheres to the Standards for Reporting Qualitative Research (SRQR) reporting guideline [23] (Additional file 1).

### Recruitment

We partnered with WIN-Gallup International Association (WIN/GIA), a reputable global research organisation, to recruit pregnant women and conduct data collection in Mexico. Altogether, 54 pregnant women from Mexico City (*n* = 26) and Toluca (*n* = 28) participated in this study. We used purposive sampling to choose participants who held negative and positive views towards maternal vaccination, and to ensure that participants were of various ages and socioeconomic status. WIN/GIA liaised with their subsidiaries in Mexico to obtain access to a panel of participants from which pregnant women were selected.

### Data collection

In-depth interviews and focus groups were conducted by WIN/Gallup International, in Spanish. Two topic guides were developed, one for in-depth interviews and another for focus groups. Topic guides were developed to explore participants’ general understanding and experience of the maternal vaccination programme, their reasons for deciding to accept or refuse vaccination, and their understanding of the risks and benefits of maternal vaccines and vaccination in general. The interview topic guide was designed to encourage participants to give their views and opinions, and not with the intention of convincing them that they needed to vaccinate. The topic guides were developed by C.S. and P.P. with input from H.J.L., and later translated to Spanish. Audio files of focus groups and interviews were translated and transcribed directly into English. Participants were compensated for travel, subsistence and participation in the research.

### In-depth interviews

Twenty in-depth interviews were conducted with pregnant women, either face-to-face or over the phone. When face-to-face interviews were possible, data collection took place at the offices of WIN/Gallup subsidiaries in Mexico City and Toluca. The majority of interviews conducted in person were observed and supervised by C.S.

### Focus groups

Four focus groups were conducted (two per location). Each group was composed of 8 to 10 women of different ages and stages of pregnancy. Groups were split into first time pregnancies and second or higher pregnancy. All focus groups required participants to attend in person and were conducted at the local country offices of WIN/Gallup, observed and supervised by C.S.

### Data management and analyses

To ensure confidentiality, all data were anonymized. Quotations from participants were used in this study and confidentiality was maintained by using solely the codes assigned (MC for Mexico City and TL to Toluca) and we ensured participants could not be identified through contextual information. Data were stored anonymously within a secure server on password protected computers. Only co-investigators cited in the ethics approval had access to the files.

Transcripts were analysed using NVivo 11 software (QSR International, Melbourne, Australia). Authors used a deductive approach, based on comprehensive literature review, to develop a standardised coding scheme. The data were organised and coded under themes which emerged when pregnant women were surveyed about different aspects of maternal immunization.

### Ethical approval

We received approval to conduct secondary data analysis from the London School of Hygiene & Tropical Medicine ethics committee in May 2019 (LSHTM ethics ref.: 17100). For primary data collection, standard industry verbal and written consent was obtained by WIN/Gallup International Association (WIN/GIA). Participants were informed that their participation was voluntary, and they were allowed to refuse to answer any question or end the interview at any time. All participants provided authorization of the use of data for research purposes only, regardless of research institution.

## Results

Based on the different dimensions of trust associated with vaccination, the findings of this study have been categorized into four themes: Trust in public health system; Trust in the safety and efficacy of maternal immunization; Trust in government; and Trust in information.

### Trust in public health system

Participants reported experiencing barriers to accessing public maternal health care services, including maternal immunization. Most participants with the financial means reported having public and private health insurance to guarantee access to care as public services were perceived as insufficient. Some participants also experienced vaccine shortages.

“I have both government and private health insurance – they told me to keep my options open. In many places the government hospitals are saturated” (MC).

“I go to public and private [healthcare institutions]. My GP recommended public [healthcare] – it can be difficult to treat complications in a private hospital, so [one should] keep going to public if you have government insurance. But we keep thinking of the concerns with public healthcare” (TL).

“If I can’t get a place at a public hospital when in labour I will go to private” (MC).

“They (public hospital) ran out of vaccines. They tell you to come back later or try other hospitals - or you can go to a private one” (MC).

Participants described constantly navigating hard choices. Pregnant women reported lower quality of care and overcrowding in public services while some participants reported higher trust in private services.

“There are so many people there (public hospitals), you have to wait a month to get an appointment, have to wait for hours when you get one, and they are very rude to older or younger pregnant women. (…). One physician (caring) for many pregnant women” (MC).

“In terms of trust I would go to the private [healthcare clinics]” (MC).

For those who could afford it, private healthcare is a way of ensuring not just access to high quality services, but also control over their healthcare choices.

“Private services mean consistency of the doctor. Quality care, more information about the journey, tailored care (…), more control” (TL).

“Private healthcare is a good, customised service. You are allowed to have visitors whenever you want, they are very nice to you. You can see your baby whenever you want. You have one physician the whole journey” (MC).

Others openly discussed mistrust in public healthcare.

“(There are) stories about wrong operations being carried out (in public hospitals), of uncleanliness, of personal belongings being stolen, so I have lower trust (in public hospitals)” (TL).

Finally, one participant discussed intuitively distrusting public healthcare services.

“I honestly don’t trust them (public hospitals), I don’t know why” (MC).

Using private health services was also viewed as a protection against the perceived aggressions in the public health system. Many participants reported previous negative experiences with healthcare professionals (HCPs) in public services and worried about feeling safe in healthcare services. In this context, participants reported feeling responsible for making a good choice.

“(There is much) rudeness in public health services – and private isn’t like that” (TL).

“I wonder if I can trust this hospital, if it is a good choice” (MC).

When attending public services, some participants felt pushed to vaccinate while they still had concerns.

“(I need an) explanation why it will be good for me and my baby. There are some vaccines they (HCP) don’t even ask you, like tetanus (vaccine), you get them whether you want it or not” (MC).

### Trust in safety and efficacy of maternal immunization

Some participants viewed maternal vaccines as a way to protect their unborn baby against infectious diseases. Yet, those participants reported doubts over the safety and effectiveness of the vaccines even after having them.

“Vaccines are necessary, but I also fear them” (MC).

“(A pregnant woman) has to convince herself it is good for her and believe it will be effective” (TL).

“I feel vaccines are useless, but I’ll give them the benefit of doubt” (MC)

“I hope (the vaccines) will work” (MC).

In these cases, a HCP recommendation encouraging maternal vaccination was a strong driver for these participants’ decision to vaccinate. While husbands and pregnant women’s mothers were cited by participants as sources of influence and support for decision making, participants recognized the expertise of HCPs.

Many participants in this study were mistrustful of the safety of maternal vaccines, worrying that babies would be born with deformities and disabilities if they vaccinated during pregnancy. In particular, several women were concerned that the flu vaccine during pregnancy could cause serious side effects. Other participants reported doubts over the benefits of vaccinating during pregnancy, leading them to delay or refuse maternal vaccination. These participants remained unconvinced of the importance and relevance of vaccination.

“There needs to be a good reason for vaccinating” (MC).

### Trust in government

Some participants reported mistrust in vaccination campaigns, believing they were used by government to divert attention from political issues.

“Flu vaccination campaign (was used) as a smokescreen for the government” (TL).

In this context, many participants reported refusing maternal immunization or their intention to refuse immunization if offered. Vaccine-accepting participants reported that correcting misinformation could be a driver for maternal vaccination. To them, government advertising campaigns and also “breaking the myths” (MC) on vaccination could be effective to convince more pregnant women to vaccinate.

### Trust in information

Many participants reported a lack of information and also were exposed to misinformation about maternal and general vaccines. One mother reported needing more information before making a decision.

“I need to know what (the vaccine) is for, need to know the risks and benefits. (I) need more information and understanding of how necessary (the vaccine) really is” (TL).

Amidst overall lack of information, as well as being faced with circulating misinformation, many participants reported going online for their health information during pregnancy, including vaccination. They did not cite HCPs as a primary source of information, preferring to search online for answers and describe trusting what they read online, impacting their health decisions.

“Saint Google. First thing you resort to(…) We have a lot more information now” (TL).

“(I use) the internet – how to notice if baby has deformities” (MC).

“I trust the internet (for health information)” (MC).

In addition to search engines, apps (e.g. *My Embarazo,* Babycentre) and social media (Facebook, YouTube, Instagram) were reported by participants as sources of health information. Many participants mentioned Facebook groups with other pregnant women. Vaccine accepting parents also participated in Facebook groups, however in contrast, these posted information of where to go to get certain vaccines for free. Some participants reported using Instagram to aid finding and evaluating possible obstetricians.

“I go to Instagram to find a doctor with lots of experience, photos, liked by many – lots of people have gone through the process, it is a way to know (if care provided is adequate)” (MC).

## Discussion

This study explores views and attitudes of pregnant women in Mexico City and Toluca, Mexico, as they navigated the health system, searched for information and made decisions around maternal immunization. Maternal vaccine confidence was explored considering pregnant women’s interactions with public and private health services, healthcare professionals and information from different sources.

Broadly, the findings of the present study point to issues in access and quality of maternal healthcare which contribute to low levels of confidence in maternal vaccination. Our findings reveal perceptions that public health services are saturated, inefficient, and of lower quality than private health services. Health systems are relational and trust in services is a key influencer of uptake of interventions, including vaccines [24]. Another key finding was that negative interactions with HCPs were often dignity-denying and kept participants away from public maternal services. Findings indicate that a perceived lack of control over their own health choices, paired with mistreatment by HCPs, can drive wealthier patients to switch to private services, where higher levels of trust are recorded, and push low-income individuals to avoid health services altogether.

Some participants held strong doubts over the benefits of vaccination and many participants were hesitant about safety and efficacy of maternal vaccines. These concerns were also shared by pregnant women who had been vaccinated. These hesitant compliers [25], i.e., fully vaccinated individuals who still hold doubts about vaccines, are a point of concern. Those who are generally adherent can switch to delaying, cherry-picking or refusing vaccination if their questions and doubts are not addressed [25]. Given the authority placed by participants in the expertise of HCPs (and their recommendation of vaccines), more should be invested in how HCPs can better inform and contribute to patient’s vaccine literacy [26].

When faced with a decision to vaccinate, it is common to seek more information - particularly during pregnancy when information-seeking behaviours are heightened [27]. The reported lack of vaccine information from HCPs paired with a heavy use of the internet for maternal health information is an issue of concern [28]. Facebook and Google, both widely cited by study participants as key information sources, have been previously studied for their potential to propagate vaccine misinformation [29]. Findings from this study suggest that rumours and misinformation about maternal vaccines are plentiful in Mexico City and Toluca and need to be addressed. Moreover, participants reported using social media to find HCPs and as proof of quality professional status. While social networking can present opportunities to HCPs to share information and increase their public profile, it is not without risks [30, 31] such as distribution of poor-quality information [32], violation of personal-professional boundaries [33] and ethical concerns [34].

Beyond generalized mistrust in the health system, wavering maternal vaccine confidence can be compounded by mistrust in political system. Government-led vaccination campaigns, against viruses like as flu, were perceived at times as cover-up tactics to divert attention away from political problems. In this context, many participants reported refusing maternal immunization or their intention to refuse immunization if offered. Themes of historic neglect or abuse from a government or health system, particularly among minorities, have been studied elsewhere as underlying reasons for distrust in vaccines in a population [35–38]. Equally important is the overall trustworthiness of those institutions [39]. By placing the burden of distrust in vaccines only on individuals, with little attention to the responsibility of institutions to be trustworthy, trust dynamics will only be partially understood and addressed [20]. Our study findings call attention to the substantial amount of dignity-denying experiences shared by participants, which is consistent with previous reports in the literature [15, 40, 41]. If they are to be trusted, political and health systems should strive for dignity-affirming experiences for their populations who sustain the hefty burden of disease and vulnerability [21].

### Limitations

First, because this is a qualitative study, the findings are not generalizable but rather convey experiences and views of participants that may not be captured in quantitative investigations. Second, owning to our sample’s composition, our findings may not reflect the experience of indigenous populations and of those in rural areas, and their experience of access to health services are likely to be different.

## Conclusion

Over the past few decades Mexico has invested heavily in public access to maternal healthcare for its population. Optimal immunization coverage among this group is fundamental to achieving maternal health goals and to reduce neonatal and maternal morbidity and mortality. In this study, we have attempted to understand views and attitudes towards maternal immunization as pregnant women navigate the Mexican healthcare system, interact with HCPs and gather information from different sources. Maternal vaccine confidence among participants was low (even among fully vaccinated women), as was trust in public hospitals and HCPs. Pregnant women are going online for information and anti-vaccine sentiments and misinformation broadcasted online may impede health-seeking behaviour among this group. Already a vulnerable group, pregnant women and their new-borns face additional risk due to low uptake of maternal immunization. Hence, these pregnant women, who are also soon becoming parents and making vaccination decisions for their child, constitute an important target group for policymakers seeking optimal maternal as well as childhood immunization coverage, with targeted communication, trust-building and engagement strategies. Evidence-based interventions and policies are urgently needed to improve vaccine confidence and vaccine coverage in maternal vaccination in order to reduce maternal and neonatal morbidity and mortality.

## Supplementary Information

**Additional file 1.** Standards for Reporting Qualitative Research (SRQR)*.

## Data Availability

The qualitative datasets generated and/or analysed during the current study are not publicly available due to the data containing information that could compromise research participant privacy/consent but are available from the corresponding author on reasonable request.
